# Diffusion Tensor Imaging With Tract-Based Spatial Statistics Reveals White Matter Abnormalities in Patients With Vascular Cognitive Impairment

**DOI:** 10.3389/fnana.2018.00053

**Published:** 2018-06-26

**Authors:** Hua-Jun Chen, Yong-Qing Gao, Chun-Hui Che, Hailong Lin, Xin-Lin Ruan

**Affiliations:** ^1^Department of Radiology, Fujian Medical University Union Hospital, Fuzhou, China; ^2^Department of Radiology, Fuqing City Hospital, Fuqing, China; ^3^Department of Neurology, Fujian Medical University Union Hospital, Fuzhou, China; ^4^Department of Geriatrics, Fujian Medical University Union Hospital, Fuzhou, China

**Keywords:** vascular cognitive impairment, diffusion tensor imaging, subcortical ischemic vascular disease, tract-based spatial statistics, local diffusion homogeneity

## Abstract

**Purpose**: The aim of this study was to evaluate microstructural changes of major white matter (WM) tracts in patients with vascular cognitive impairment (VCI).

**Method**: Diffusion tensor imaging (DTI) data were obtained from 24 subjects with subcortical ischemic vascular disease (SIVD), including 13 subjects with VCI-no dementia (VCIND) and 11 subjects with normal cognition (as a control group). A tract-based spatial statistics approach was performed to investigate WM microstructure in VCIND by integrating multiple indices including fractional anisotropy (FA) and mean diffusivity (MD), which are intra-voxel metrics, and local diffusion homogeneity (LDH), which is an inter-voxel metric.

**Results**: The VCIND group had decreased FA and increased MD values throughout widespread WM areas predominately in the corpus callosum, bilateral internal capsule/corona radiata/posterior thalamic radiation/inferior fronto-occipital fasciculus and right inferior/superior longitudinal fasciculus. There was a slight discrepancy between the distribution of areas with decreased FA and LDH. The FA, MD and LDH values were significantly correlated with cognitive test results. According to a WM tract atlas, 10 major tracts were identified as tracts of interest in which three diffusion metrics simultaneously differed between groups, including bilateral anterior thalamic radiation, forceps minor, right corticospinal tract, bilateral inferior fronto-occipital fasciculus, left inferior and superior longitudinal fasciculus, and bilateral uncinate fasciculus. Receiver operating characteristic (ROC) analysis demonstrated the feasibility of using diffusion metrics along the forceps minor and left anterior thalamic radiation for separating two groups.

**Conclusion**: The results suggest WM microstructural abnormalities contribute to cognitive impairments in SIVD patients. DTI parameters may be potential biomarkers for detecting VCIND from SIVD.

## Introduction

Vascular cognitive impairment (VCI) is defined as cognitive deficits attributable to various cerebrovascular causes (such as small and large vessel diseases), which can be classified into three subtypes: vascular cognitive impairment-no dementia (VCIND), vascular dementia, and mixed primary neurodegenerative dementia (usually Alzheimer’s disease) with vascular dementia (Moorhouse and Rockwood, 2008). VCI is attracting increasing attention due to its high prevalence (Moorhouse and Rockwood, 2008) and high treatment cost (Rockwood et al., 2002). Given the potential preventability of VCI (especially at the early stage; Middleton et al., 2008; Moorhouse and Rockwood, 2008), many studies have been performed to identify new biomarkers that can provide early diagnosis and be used to monitor progression of disease as well as assess therapeutic effects in clinical trials.

White matter (WM) involvement in VCI has been demonstrated by many neuroimaging studies. For example, magnetic resonance spectroscopy studies have demonstrated WM metabolic changes such as the reduced N-acetylaspartate levels (Chen et al., 2016) in VCI patients, which is thought to be associated with altered cognitive status such as the executive dysfunction (Gasparovic et al., 2013). Studies utilizing diffusion tensor imaging (DTI) have revealed that VCIND patients have impaired microstructural integrity, which is reflected by decreased fractional anisotropy (FA), throughout widespread WM areas (Zhou et al., 2011). Furthermore, correlation analyses have consistently shown that DTI metrics are associated with cognitive performance among patients with small vessel disease (SVD, one of most common causes for VCI; Kim et al., 2011; Zhou et al., 2011; Tuladhar et al., 2015). More recently, several DTI studies, which utilizes the fiber tracking technique and graph theoretical analysis, have highlighted disruption of structural WM networks (e.g., reduced connection strengths and network efficiency) in SVD (Tuladhar et al., 2016a), which is considered as an important neural substrate of cognitive impairment (Lawrence et al., 2014) and a predictor of disease progression (Tuladhar et al., 2016b).

The present study aimed to evaluate microstructural changes of major WM tracts related to small vessel disease using DTI and tract-based spatial statistics (TBSS) analysis in patients with VCI. TBSS is a method for voxel-based analysis of WM diffusion data (Smith et al., 2006), in which diffusion metric maps from each subject are projected onto a skeleton located at the center of major WM pathways and then compared across groups. It minimizes registration error compared with voxel-level analysis based on statistical parametric mapping and personal evaluation bias compared with region of interest (ROI) level analysis. The TBSS framework also avoids the necessity of choosing a spatial smoothing procedure during voxel-based analysis and is thought to improve sensitivity, objectivity, and interpretability when it is applied to multi-subject diffusion datasets (Smith et al., 2006). To the best of our knowledge, few TBSS studies have been performed to determine directly diffusion measurement differences between subcortical ischemic vascular disease (SIVD) patients with and without mild cognitive impairment. In addition, for the first time, both intra-voxel and inter-voxel diffusion metrics were simultaneously examined in the VCIND patients to characterize more fully WM microstructural changes.

## Materials and Methods

### Participants

This study was approved by the Ethics Committee of Fujian Medical University Union Hospital. Twenty-four subjects, who provided written informed consent, were recruited from a stroke/transient ischemic attack clinic. SIVD were diagnosed if the subjects had the subcortical WM hyperintensity on T2-FLAIR imaging with at least one lacunar infarct, in accordance with the criteria suggested by Galluzzi et al. (2005). Detailed information on patient history and clinical complaints was collected. All patients underwent comprehensive physical, neurological, and neuropsychological assessments as well as laboratory examinations to exclude systemic or other neurological diseases.

Exclusion criteria were as follows: (1) cortical and/or cortico-subcortical non-lacunar territorial infarcts and watershed infarcts or hemorrhages; (2) WM lesions with specific causes (e.g., multiple sclerosis, sarcoidosis, brain irradiation and metabolic disorder); (3) signs of normal pressure hydrocephalus or alcoholic encephalopathy; (4) neurodegenerative disease such as Alzheimer’s disease and Parkinson’s disease; (5) other psychiatric comorbidity or severe cognitive impairment (inability to perform neuropsychological tests); and (6) severe claustrophobia and contraindications to MRI.

The inclusion criteria for the VCIND group were as follows: (a) subjective cognitive complaints reported by the participant or caregiver; (b) objective cognitive impairments but failure to meet the Diagnostic and Statistical Manual of Mental Disorders, fourth edition (DSM-IV) criteria for dementia, mini-mental state examination (MMSE) score > 24, and clinical dementia rating scale (CDR) score = 0.5; (c) normal or almost normal general cognitive functioning and normal activities of daily living or slight impairment in instrumental activities of daily living; and (d) Montreal Cognitive Assessment (MoCA) score < 26 (Freitas et al., 2012; Xu et al., 2014). SIVD patients were subdivided into two groups. One group had normal cognition (*n* = 11, as a control group), and the other group was the VCIND group (*n* = 13). No significant differences were found with respect to age, gender and education between the two groups (Table 1).

**Table 1:** Demographic characteristics of the subjects.

	Control group (*n* = 11)	VCIND group (*n* = 13)	*P* value	
Age (years)	66.4 ± 8.5	67.1 ± 8.2	0.832
Sex (male/female)	7/4	8/5	0.916 (*χ*^2^-test)
Education level (years)	10.6 ± 3.4	9.1 ± 3.6	0.306
MoCA score	27.1 ± 0.8	18.4 ± 5.7	<0.001

*VCIND, vascular cognitive impairment-no dementia; MoCA, Montreal Cognitive Assessment*.

### MRI Data Acquisition

MR images were acquired on a 3-Tesla MRI scanner (Discovery MR750, General Electric Healthcare). Routine T1-weighted, T2-weighted and T2-FLAIR images were obtained to exclude possible lesions specified in the exclusion criteria. DTI data were obtained using a single-shot diffusion-weighted echo planar imaging sequence with the following parameters: TR/TE = 8325/87.6 ms, FOV = 256 × 256 mm^2^, matrix size = 128 × 128, slice thickness = 4 mm, number of slices = 35, number of diffusion gradient directions = 40 and *b*-value = 1000 s/mm^2^.

### DTI Data Processing

DTI data were processed using FMRIB’s diffusion toolbox (FSL 4.0)^1^. Eddy current-induced distortion and motion artifacts in the DTI dataset were corrected using affine alignment of each diffusion-weighted image to the b0 image. After skull-stripping, the diffusion tensor matrix for each subject was calculated. Thus, three pairs of eigenvalues and eigenvectors were obtained from the diffusion tensor matrix. Then, the intra-voxel metrics, including FA and mean diffusivity (MD), were calculated based on the three eigenvalues. The inter-voxel metric, local diffusion homogeneity (LDH, the predefined neighborhood = 27 voxels), was also calculated according to previous studies (Gong, 2013; Zhuo et al., 2016). The LDH calculation was completed using a pipeline toolbox called PANDA (Cui et al., 2013)^2^.

A TBSS approach was performed to investigate changes in diffusivity parameters along WM tracts. After FA maps were computed for all subjects using the FMRIB diffusion toolbox, FA data from each participant were further processed and analyzed using the TBSS tool available in FSL^3^. The TBSS procedure was as follows. FA images were normalized to the standard FMRIB58 FA template using the nonlinear registration algorithm in FSL. The normalized FA images were averaged to create the mean FA map. The mean FA map was the input to the tract skeleton generation step. The skeleton of a tract was a single line (or surface) running down the center of this tract. For FA skeleton generation, a FA threshold of 0.2 was used to exclude voxels that were primarily gray matter or cerebrospinal fluid. After FA skeleton generation, an individual subject’s FA was projected onto the FA skeleton. We use non-parametric permutation method in FSL (FSL randomize procedure) to test FA differences between groups. WM abnormalities were investigated using the threshold-free cluster enhancement at *P* value < 0.05 (5000 permutations) fully corrected for multiple comparisons. A similar tract-based analysis procedure was applied to the MD and LDH images.

### Correlation Analysis

The WM areas with between-group diffusion parameter differences were defined as ROI. The mean FA, MD and LDH values in the ROI were calculated. Then, Spearman correlation analysis was used to assess the relationships between diffusion metrics and cognitive performance.

**Figure 1:**
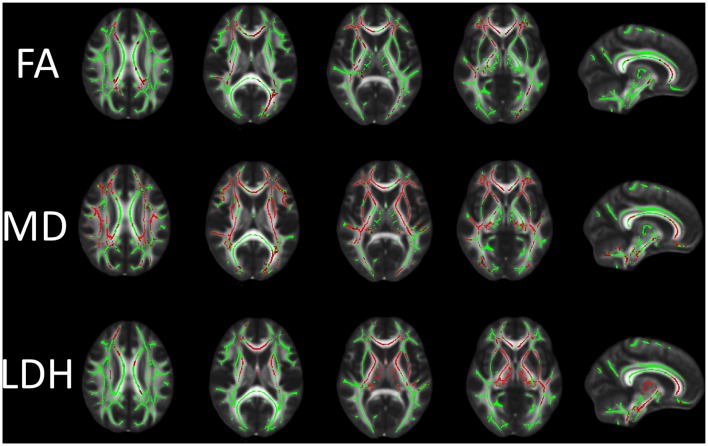
Differences in fractional anisotropy (FA), mean diffusivity (MD) and local diffusion homogeneity (LDH) between the vascular cognitive impairment (VCI) no dementia (VCIND) and control groups. Green indicates the FA skeleton with a threshold of 0.2, which highlights the tracts used in the comparison. Red indicates the regions with decreased FA and LDH values and increased MD values in the VCIND group.

**Figure 2:**
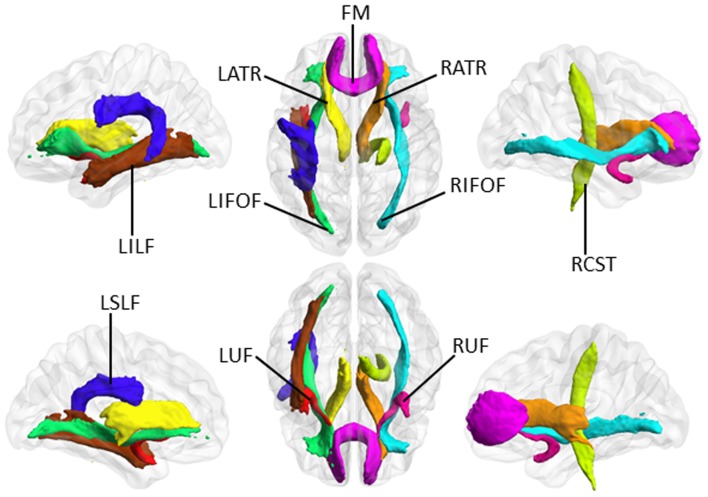
The selected major tracts according to the JHU white-matter tractography atlas (https://fsl.fmrib.ox.ac.uk/fsl/fslwiki/Atlases). In these white matter (WM) tracts, TBSS analysis demonstrated that VCIND patients had significant changes in all three diffusion parameters. “R” and “L” indicate right and left side, respectively. LATR, left anterior thalamic radiation; RATR, right anterior thalamic radiation; RCST, right corticospinal tract; FM, forceps minor; LIFOF, left inferior fronto-occipital fasciculus; RIFOF, right inferior fronto-occipital fasciculus; LILF, left inferior longitudinal fasciculus; LSLF, left superior longitudinal fasciculus; LUF, left uncinate fasciculus; RUF, right uncinate fasciculus.

In addition, according to the JHU white-matter tractography atlas^4^, 10 major WM tracts were selected as tracts of interest (TOI) because TBSS analysis demonstrated that VCIND patients had significant changes in all three diffusion parameters along these WM tracts (see “Results” section, Figures 1, 2). The mean FA, MD and LDH values along these TOI were calculated and then correlations with the neurocognitive test scores were assessed.

### Exploratory Classification Analysis

To examine diffusion parameters in the left anterior thalamic radiation and forceps minor as a possible diagnostic biomarker, receiver operating characteristic (ROC) analysis was used to assess the discrimination performance of diffusion metrics between the two groups. The area under the ROC curve (AUC) was calculated using SPSS version 16.0 (SPSS Inc., Chicago, IL, USA). The left anterior thalamic radiation and forceps minor were selected as target tracts since all three diffusion measurements of these two tracts were found to be significantly correlated with cognitive performance (see “Results” section, Table 2).

**Table 2:** Correlations between diffusion metrics in major white matter (WM) tracts and the cognitive test score.

Fiber tract	FA value vs. MoCA score		MD value vs. MoCA score		LDH value vs. MoCA score	
	CC	*P* value	CC	*P* value	CC	*P* value
**LATR**	0.452	0.026 **(0.045)**	−0.579	0.003 **(0.031)**	0.566	0.004 **(0.032)**
RATR	0.512	0.011 **(0.032)**	−0.496	0.013 **(0.032)**	0.433	0.034 (0.052)
RCST	0.337	0.107 (0.120)	−0.447	0.028 **(0.045)**	0.392	0.058 (0.073)
**FM**	0.493	0.014 **(0.032)**	−0.496	0.013 **(0.032)**	0.548	0.006 **(0.032)**
LIFOF	0.465	0.022 **(0.041)**	−0.503	0.012 **(0.032)**	0.362	0.082 (0.099)
RIFOF	0.501	0.013 **(0.031)**	−0.517	0.009 **(0.031)**	0.401	0.052 (0.068)
LILF	0.533	0.007 **(0.032)**	−0.474	0.019 **(0.038)**	0.328	0.118 (0.127)
LSLF	0.451	0.027 **(0.045)**	−0.491	0.014 **(0.032)**	0.065	0.762 (0.763)
LUF	0.407	0.048 (0.066)	−0.525	0.008 **(0.031)**	0.346	0.098 (0.113)
RUF	0.420	0.041 (0.059)	−0.497	0.013 **(0.032)**	0.179	0.403 (0.417)

*LATR, left anterior thalamic radiation; RATR, right anterior thalamic radiation; RCST, right corticospinal tract; FM, forceps minor; LIFOF, left inferior fronto-occipital fasciculus; RIFOF, right inferior fronto-occipital fasciculus; LILF, left inferior longitudinal fasciculus; LSLF, left superior longitudinal fasciculus; LUF, left uncinate fasciculus; RUF, right uncinate fasciculus; CC, correlation coefficient. The numbers in the bracket indicate the corrected* P* values. Here, the false-discovery rate (FDR) correction is applied for multiple comparisons (*n* = 30). Bold numbers indicate a corrected *P* value < 0.05. It is noteworthy that all three diffusion parameter values in LATR and FM were significantly correlated with the cognitive test score*.

## Results

The WM regions with significant between-group differences in diffusion metrics are shown in Figure 1. Compared with the control group, the VCIND group had significantly decreased FA values in widespread WM areas predominately in the corpus callosum, right internal capsule, bilateral corona radiata, bilateral posterior thalamic radiation (including the optic radiation), left cingulum, bilateral inferior longitudinal fasciculus, right superior longitudinal fasciculus and bilateral inferior fronto-occipital fasciculus. The distribution of areas with increased MD was similar but more extensive than that of areas with decreased FA. The areas with increased MD were mainly located in the corpus callosum, left cingulum, bilateral corticospinal tract, cerebellar peduncle, internal and external capsule, corona radiata, posterior thalamic radiation (including the optic radiation), inferior/superior longitudinal fasciculus and bilateral inferior fronto-occipital fasciculus. Compared with controls, VCIND patients showed decreased LDH mainly in the corpus callosum, left posterior thalamic radiation (including the optic radiation), inferior/superior longitudinal fasciculus, bilateral corticospinal tract, internal and external capsule, corona radiata, and inferior fronto-occipital fasciculus. It was notable that there was a slight discrepancy between the distribution of areas with decreased FA and LDH (e.g., decreased LDH was negligible in the bilateral occipital lobes). This was expected since previous reports indicated that intra-voxel and inter-voxel metrics could yield discrepant findings even under the same pathological condition (Gong, 2013; Zhuo et al., 2016).

Based on the above results of TBSS analysis, 10 major WM tracts, including the bilateral anterior thalamic radiation, forceps minor, right corticospinal tract, bilateral inferior fronto-occipital fasciculus, left inferior and superior longitudinal fasciculus, and bilateral uncinate fasciculus, were identified as TOI according to the JHU white-matter tractography atlas^5^. During TBSS analysis, FA, MD and LDH alterations simultaneously involved these TOI. Figure 2 illustrates these 10 major WM tracts.

**Figure 3:**
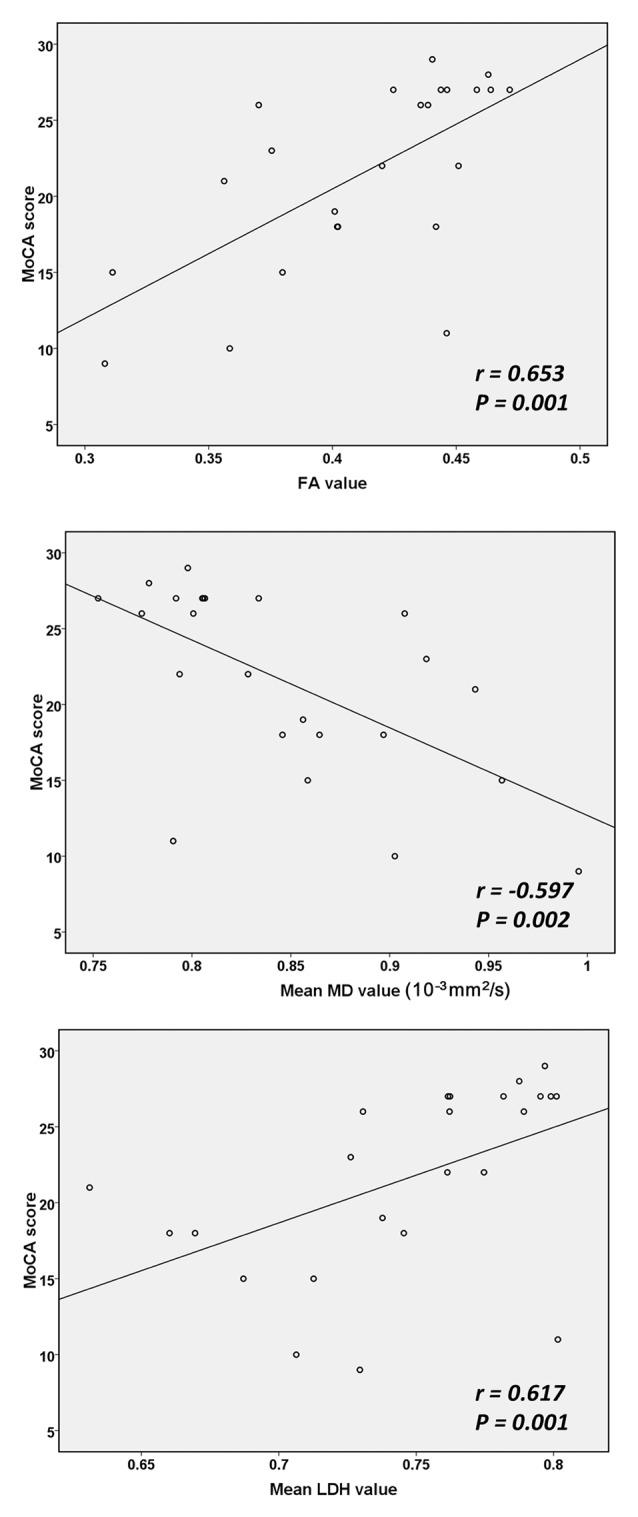
Correlations between diffusion measurements and cognitive test scores. MoCA, Montreal Cognitive Assessment. The mean FA, MD and LDH values in the regions with between-group diffusion metric differences were calculated and then correlations with MoCA scores were assessed.

Based on the ROI selection, correlation analyses showed that the WM, FA and LDH values were positively correlated with the results of cognitive tests among SIVD patients, whereas the WM MD value was negatively correlated with the cognitive test scores (Figure 3). Based on the TOI selection, correlation analyses demonstrated that the mean FA values along several major WM tracts, including the bilateral anterior thalamic radiation, forceps minor, bilateral left inferior fronto-occipital fasciculus and left inferior/superior longitudinal fasciculus, were positively correlated with the cognitive test scores (Table 2). The mean MD values along all 10 TOI were negatively correlated with cognitive performance. Finally, the LDH values were positively correlated with the results of cognitive tests along only two TOIs (the left anterior thalamic radiation and forceps minor).

**Figure 4:**
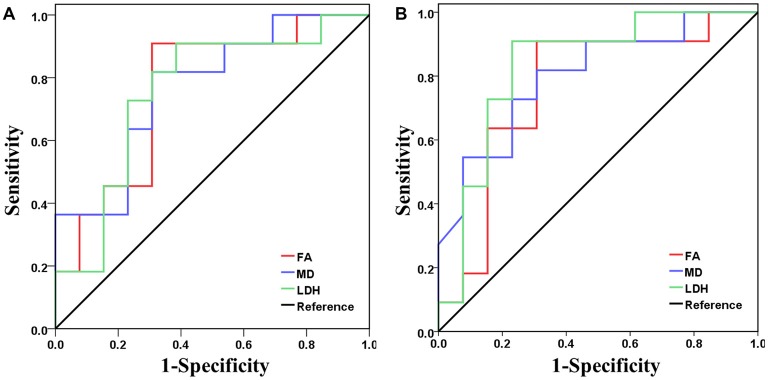
Receiver operating characteristic (ROC) curves for FA (red), MD (blue) and LDH (green) in separating the two groups. Panels **(A,B)** show results when the diffusion metric along the forceps minor and left anterior thalamic radiation, respectively, was used as the discrimination index.

Based on the above correlation analysis, we found that all three diffusion metrics along the left anterior thalamic radiation and forceps minor were significantly correlated with the cognitive test scores. Thus, these two WM tracts were selected as target tracts for the following analysis. ROC analysis demonstrated the feasibility of using FA, MD and LDH to distinguish the VCIND patients from the controls (Figure 4). The FA (AUC = 0.762), MD (AUC = 0.769) and LDH (AUC = 0.755) along the forceps minor had moderate discrimination potential. In addition, the FA (AUC = 0.762), MD (AUC = 0.801) and LDH (AUC = 0.832) along the left anterior thalamic radiation showed moderate potential for separating the two groups.

## Discussion

By measuring the motion of water, DTI provides a powerful and unique tool for examining the organization of the human brain and structural abnormalities related to various neuropsychological disorders. In this study, the TBSS approach was applied to DTI data, and altered microstructural properties of major WM tracts were identified in VCIND patients by measuring both intra-voxel and inter-voxel diffusion metrics. This finding supports the contention that the key underlying pathology in VCIND involves WM structural abnormalities (Zhou et al., 2011; Lin et al., 2015), which may contribute to a disconnection syndrome and correlate with loss of cognitive function (López-Gil et al., 2014; Tuladhar et al., 2016a). Furthermore, consistent with previous studies (Kim et al., 2011; Zhou et al., 2011; Tuladhar et al., 2015), we found a correlation between distinct diffusion metrics and cognitive test results, which suggests that intra-voxel and inter-voxel diffusion characteristics may serve as the diagnostic biomarker of VCIND. Moreover, the results of ROC analysis suggest moderate classification efficiency between groups using diffusion metrics along the left anterior thalamic radiation and forceps minor.

In this study, both intra-voxel and inter-voxel diffusion metrics were examined simultaneously to characterize comprehensively WM microstructural changes. FA represents a normalized ratio of diffusion directionality, which reflects the degree of alignment of cellular structures within fiber tracts and their structural integrity. MD measures bulk mobility of water molecules, which reflects overall whole molecule diffusion level and diffusion resistance. Although the specific cellular mechanisms underlying changes of these two intra-voxel diffusion measures remain unclear in VCIND, our findings of decreased FA and increased MD may be explained by loss of axonal fibers, demyelination, or both. Support for this hypothesis comes from previous reports in which diffuse WM changes with loss of myelin and axonal abnormalities have been well documented in almost all subtypes of vascular dementia (Zhou et al., 2008; Kalaria, 2016). In the current study, axial diffusivity (AD) and radial diffusivity (RD), which reflect axon and myelin pathologies, respectively (Song et al., 2003), were also examined (see Supplementary Figure S1). Increased axial and radial diffusivities were detected in most WM tracts with altered FA and MD, which suggests both axon and myelin pathologies in VCIND. In addition to FA and MD, the inter-voxel diffusion metric-LDH, which quantifies local coherence of water molecule diffusion within a neighborhood and reflects microstructural coherence of underlying WM fibers, was also compared between groups. Decreased LDH may represent loss of local coherence of fiber orientation, myelination, diameter, or density along the WM tracts affected by VCI (Gong, 2013; Zhuo et al., 2016). LDH is thought to provide additional information for understanding pathological processes in WM tracts and may serve as a complementary marker for neurological diseases (Gong, 2013; Zhuo et al., 2016). Our results indicated a correlation between cognitive function and LDH in SIVD, and the moderate diagnostic capability of the LDH metric suggests its potential as biomarker for detecting VCIND.

Previous studies have suggested that cognitive disturbances in subjects with SIVD are closely related to microstructural changes in multiple WM fibers connecting distinct cortical and subcortical regions (Zhou et al., 2008; Tuladhar et al., 2015). Our findings further verify this relationship. Moreover, it was noted that almost all WM tracts (such as the forceps minor, bilateral anterior thalamic radiation and inferior fronto-occipital fasciculus), in which diffusion measures were correlated with cognitive test scores among SIVD patients, involved WM of the frontal lobe. Selective involvement of frontal tracts may represent the neural substrates that underlie the principal component of VCI (executive dysfunction), given the prefrontal executive function (Goldman-Rakic, 1996; Fuster, 2001) and the important role of these connecting tracts in constituting executive circuits in the brain. For example, the anterior thalamic radiation carrying nerve fibers between the prefrontal cortex and the mediodorsal and anterior thalamic nuclei serves as part of the fronto-striato-thalamic loops with important implications in executive function (Masterman and Cummings, 1997; Mamah et al., 2010). The forceps minor is considered a substantial circuit that connects the bilateral regions of the anterior default mode network (van den Heuvel et al., 2009), and deactivation of this network plays an essential role in executive function (Fox et al., 2005). The inferior fronto-occipital fasciculus is thought to play important roles in reading, attention, executive function and visual processing (Catani and Thiebaut de Schotten, 2008; Wu et al., 2016). Finally, ROC analysis supports utility of diffusion metrics within the two frontal tracts (the left anterior thalamic radiation and forceps minor) in detecting VCIND. Thus, measurement of diffusion properties along frontal WM tracts may offer great potential as a biomarker for early diagnosis of VCI.

This study has several limitations. First, as a preliminary study, the sample size was small. Evaluation of a large cohort is required to strengthen statistical power and validate our findings. Second, we used a suboptimal DTI sequence with a relatively large slice thickness. Third, because of the lack of a healthy control group, we were unable to assess whether diffusion measures were abnormal in the patient sample. Fourth, this study investigated correlations between global cognitive function evaluated by the MoCA and WM microstructural changes in VCI patients. A more comprehensive assessment of cognition is needed to elucidate the mechanisms underlying specific neuropsychological dysfunction (e.g., impaired executive function) among VCIND patients. Fifth, a longitudinal study is warranted to test whether DTI measures can predict development of cognitive impairment in patients with SIVD.

In summary, VCIND patients demonstrated both intra-voxel and inter-voxel diffusion alterations along major WM tracts, which indicates an important role for changes in WM microstructure in neural substrates of VCI. In addition, the significant findings indicate DTI metrics that are sensitive to neuropathology related to VCI and provide further evidence that intra-voxel and inter-voxel diffusion characteristics may serve as a biomarker of disease.

## Author Contributions

H-JC, Y-QG and C-HC conceived and designed the study, acquired and analyzed the data, and wrote the manuscript. HL and X-LR contributed to data analysis. All authors have read and approved the manuscript.

## Conflict of Interest Statement

The authors declare that the research was conducted in the absence of any commercial or financial relationships that could be construed as a potential conflict of interest.

## Footnotes

^1^http://www.fmrib.ox.ac.uk/fsl

^2^http://www.nitrc.org/projects/panda

^3^http://fsl.fmrib.ox.ac.uk/fsl/fslwiki/

^4^https://fsl.fmrib.ox.ac.uk/fsl/fslwiki/Atlases

^5^https://fsl.fmrib.ox.ac.uk/fsl/fslwiki/Atlases

## Supplementary Material

FIGURE S1 Differences in axial diffusivity (AD) and radial diffusivity (RD) between the VCIND and control groups. Green indicates the FA skeleton with a threshold of 0.2, which highlights the tracts used in the comparison. Red indicates the regions with increased AD and RD values in the VCIND group.

## Abbreviations

AUC: area under receiver operating characteristic curve
CDR: clinical dementia rating
DTI: diffusion tensor imaging
FA: fractional anisotropy
LDH: local diffusion homogeneity
MD: mean diffusivity
MMSE: mini-mental state examination
MoCA: Montreal Cognitive Assessment
ROC: receiver operating characteristic
ROI: region of interest
SIVD: subcortical ischemic vascular disease
SVD: small vessel disease
TBSS: tract-based spatial statistics
TOI: tracts of interest
VCI: vascular cognitive impairment
VCIND: vascular cognitive impairment-no dementia
WM: white matter.
