# Supplementary figures and images for: Diffusion Tensor Imaging With Tract-Based Spatial Statistics Reveals White Matter Abnormalities in Patients With Vascular Cognitive Impairment
Source: Front Neuroanat. 2018 Jun 26;12:53. doi: 10.3389/fnana.2018.00053 (PMC6028522; doi:10.3389/fnana.2018.00053)

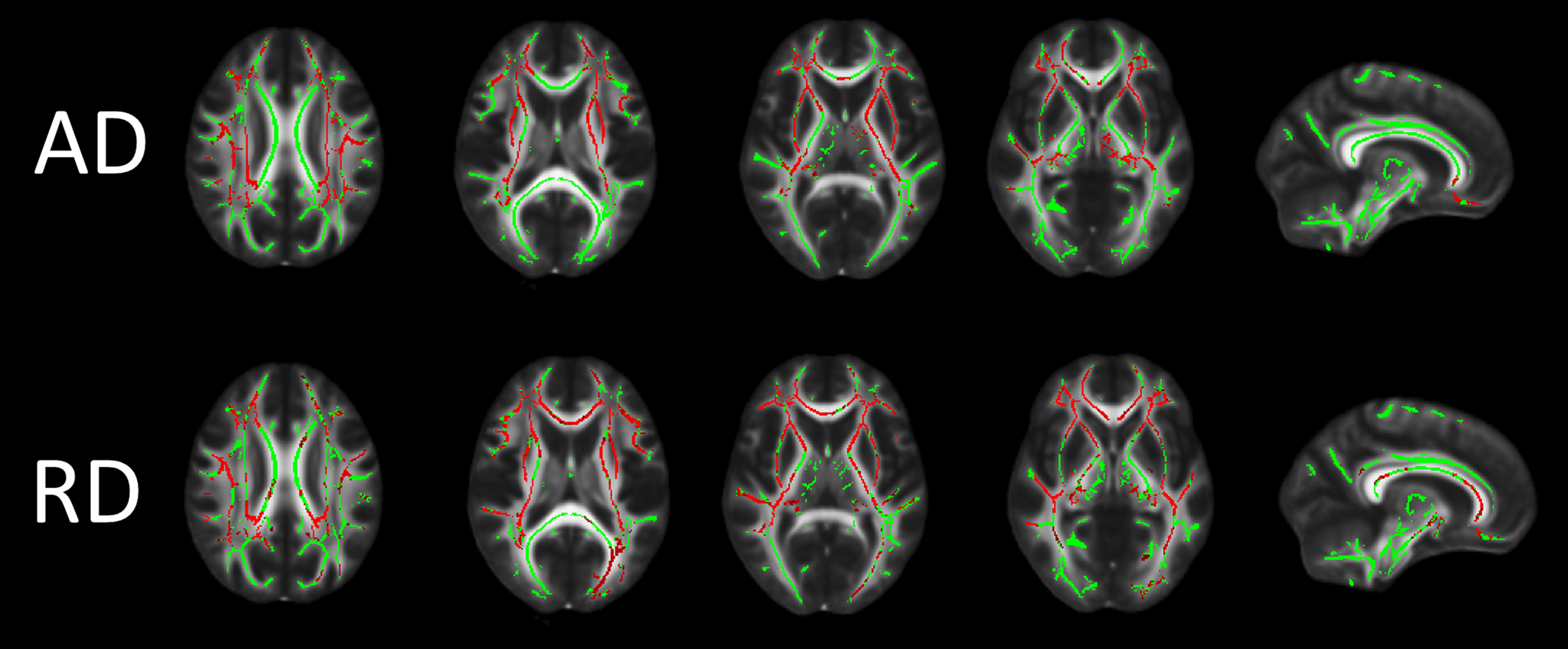

Supplement: FIGURE S1 — Differences in axial diffusivity (AD) and radial diffusivity (RD) between the VCIND and control groups. Green indicates the FA skeleton with a threshold of 0.2, which highlights the tracts used in the comparison. Red indicates the regions with increased AD and RD values in the VCIND group. [file Image_1.TIF]
